# In Situ Nano-Indentation of a Gold Sub-Micrometric Particle Imaged by Multi-Wavelength Bragg Coherent X-ray Diffraction

**DOI:** 10.3390/ma15186195

**Published:** 2022-09-06

**Authors:** Florian Lauraux, Stéphane Labat, Marie-Ingrid Richard, Steven J. Leake, Tao Zhou, Oleg Kovalenko, Eugen Rabkin, Tobias U. Schülli, Olivier Thomas, Thomas W. Cornelius

**Affiliations:** 1 Aix Marseille University, Université de Toulon, CNRS, IM2NP, 13397 Marseille, France; 2 ID01/ESRF–The European Synchrotron, 71 Avenue des Martyrs, 38000 Grenoble, France; 3 Center for Nanoscale Materials, Argonne National Laboratory, 9700 S Cass Ave, Lemont, IL 60439, USA; 4 Department of Materials Science and Engineering, Technion–Israel Institute of Technology, Haifa 3200003, Israel

**Keywords:** in situ nano-indentation, Bragg coherent X-ray diffraction imaging (BCDI), plasticity

## Abstract

The microstructure of a sub-micrometric gold crystal during nanoindentation is visualized by in situ multi-wavelength Bragg coherent X-ray diffraction imaging. The gold crystal is indented using a custom-built atomic force microscope. A band of deformation attributed to a shear band oriented along the (221) lattice plane is nucleated at the lower left corner of the crystal and propagates towards the crystal center with increasing applied mechanical load. After complete unloading, an almost strain-free and defect-free crystal is left behind, demonstrating a pseudo-elastic behavior that can only be studied by in situ imaging while it is invisible to ex situ examinations. The recovery is probably associated with reversible dislocations nucleation/annihilation at the side surface of the particle and at the particle-substrate interface, a behavior that has been predicted by atomistic simulations. The full recovery of the particle upon unloading sheds new light on extraordinary mechanical properties of metal nanoparticles obtained by solid-state dewetting.

## 1. Introduction

The mechanical properties of low-dimensional materials have been demonstrated to differ significantly from those of their bulk counterparts, as already reported in the 1950s by Herring and Galt [1] as well as by Brenner [2] about microwires showing elevated elastic limits. Pioneered by Uchic et al. in the early 2000s [3,4], numerous compression tests on micropillars of face-centered cubic (*fcc*) metals prepared by focused ion beam (FIB) milling revealed a yield strength that increases for specimens with decreasing size reaching the ultimate limit of the respective materials for defect-scarce nanowires/nanostructures. While this trend, which became known in the literature as “smaller is stronger”, is well-established nowadays, the nucleation of dislocations in defect-free low-dimensional materials is still not fully understood. It is worth noting that the increased elastic limit results in an elevated elastic strain energy that is contained in a mechanically deformed nanostructure and that is (partially) released during the nucleation of the first defects. These defect avalanches are the reason for pop-in events in nano-indentation. 

The experimental data on nano-mechanical tests typically show a large scatter that is caused by the sample microstructure, which was often overlooked. A complete picture of the size-effects on the strength of nanomaterials can thus only be painted by studying the relationship between the microstructure and the mechanical response. Dedicated quantitative mechanical testing rigs coupled in situ with imaging techniques (such as scanning and transmission electron microscopy) or synchrotron X-ray diffraction methods (Laue microdiffraction and Bragg coherent X-ray diffraction) have been developed in the recent past to overcome these shortcomings and visualize the initial microstructure of a specimen and follow its evolution during mechanical deformation [5,6,7,8,9,10]. Using these novel in-situ tools, extraordinary mechanical behaviors were reported for nanostructures such as pseudo-elastic twinning–detwinning for gold nanowires cyclically deformed in compression and in tension [8] or the liquid-like behavior of crystalline silver nanoparticles under compression [9].

In this work, we follow the microstructure of a gold sub-micrometric crystal during nano-indentation using multi-wavelength (mw) Bragg coherent X-ray diffraction imaging (BCDI) [11,12] in combination with the custom-built scanning force microscope SFINX used as an indenter [13,14]. Coherent X-ray diffraction imaging (CDI) is a lens-less microscopy technique where the real space image is retrieved from the high-resolution reciprocal space data using computational inversion algorithms. In Bragg geometry, the retrieved phase is directly related to the displacement field projected onto the diffraction vector [15]. Bragg CDI (BCDI) has been demonstrated to be highly sensitive to any kind of defect and dislocation, where the simplest dislocation configurations in *fcc* metals can be directly identified from their signatures in the 3D Bragg coherent X-ray diffraction patterns (BCDPs) [16]. Semi in situ nano-indentation on sub-micrometric Au crystals imaged by BCDI demonstrated the capabilities of this novel combination to detect and image a prismatic dislocation loop, which was induced by the mechanical loading and trapped inside the crystal after unloading [14]. In classical BCDI, the sample is rocked so that the incident angle of the X-ray beam varies. Any movements of the sample stage motors induce detrimental vibrations that lead to damage to the nanostructure in contact with a nano-indenter. These vibration issues are circumvented here by mw-BCDI, where the photon energy of the incident X-ray beam is varied instead, thus probing the reciprocal space in the vicinity of a selected Bragg peak [12,17,18]. In this work, we evidence a pseudo-elastic behavior of a gold sub-micrometer crystal where a band of defects on a (221) lattice plane is induced. It propagates towards the crystal center with increasing load and disappears, leaving a defect-free crystal behind once completely unloaded.

## 2. Materials and Methods

Single-crystalline gold particles were obtained by solid state dewetting of a 30 nm thin Au film that was deposited by electron-beam evaporation on a *c*-plane oriented polished sapphire substrate that was patterned with ~2 µm-sized holes in a marked 50 µm pitch grid [19]. The samples were annealed in an ambient atmosphere at 900 °C for 24 h, leading to the agglomeration of the patterned thin film and the formation of single-crystalline faceted Au particles. The substrate patterning eventually results in a regular array of Au crystals with a single isolated particle in the center of each square [12,14]. All crystals exhibit the same well-defined out-of-plane orientation with the Au [111] direction being parallel to the (0001) sapphire surface normal.

Multi-wavelength Bragg coherent X-ray diffraction imaging (mw-BCDI) of the specular Au **111** Bragg peak was performed at the ID01 beamline at the European Synchrotron ESRF in Grenoble (France) [20]. The photon energy of the incident focused X-ray beam was scanned from 8.75 to 9.25 keV in steps of 2 eV. Simultaneously, the scattering angle 2*θ* of the 2D MAXIPIX pixel detector used for recording the BCDPs was varied by −/+ 1° in steps of 8 millidegrees [12]. The incident intensity *I*_0_ was maintained constant by readjusting the undulator gaps every 5 steps (corresponding to 10 eV). Further experimental details, including the algorithmic procedure used for recovering the direct-space images are given in the Supplementary Material. The reconstructed direct-space images have a voxel size of 13 × 13 × 13 nm^3^.

For applying nano-mechanical stimuli in situ, the custom-built scanning force microscope “SFINX” was mounted on the diffractometer [13,14]. Prior to nano-indentation, the SFINX-tip was centered on the Au (111) top facet of a gold crystal by tapping mode AFM imaging. The selected Au crystal with the SFINX-tip hovering above was located by scanning X-ray diffraction mapping of the Au **111** Bragg reflection of an extended area of several tens of square micrometers of the sample recording the diffraction yield [21,22]. The selected crystal was then loaded mechanically with the SFINX-tip by applying a constant force rate of 10 nN/s. A slice of the 3D Bragg diffraction pattern was monitored simultaneously at a 1 Hz rate. At six pre-defined loads, the loading process was interrupted and the indented crystal was imaged in situ by mw-BCDI. Before loading and after complete unloading, the Au crystal was imaged by BCDI using standard rocking curves in an angular range of ± 1° in steps of 0.005 degrees.

## 3. Results

**Figure 1:**
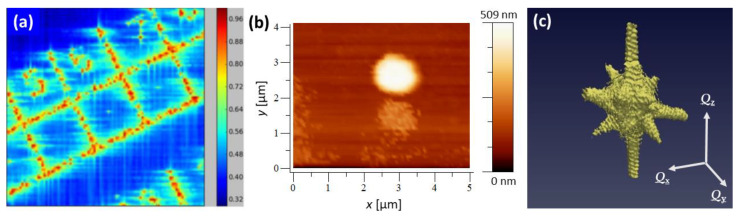
(**a**) Scanning X-ray diffraction map in diffraction contrast at the Au 111 Bragg peak of the patterned samples surface. (**b**) In situ AFM image and (**c**) 3D mw-Bragg coherent X-ray diffraction pattern of a gold crystal.

Figure 1a displays a scanning X-ray diffraction map in diffraction contrast at the Au **111** Bragg peak of the patterned sample surface. Single Au crystals are observed in the center of 50 µm large squares. An AFM topography image of a gold sub-micrometric crystal located in such a square, measured at the synchrotron end-station, is presented in Figure 1b. The corresponding 3D mw-BCDP of the Au **111** Bragg peak is displayed in Figure 1c. The 3D Bragg pattern exhibits straight streaks along the facet normals with well-pronounced size fringes as expected for a defect-free and weakly strained object. 

**Figure 2:**
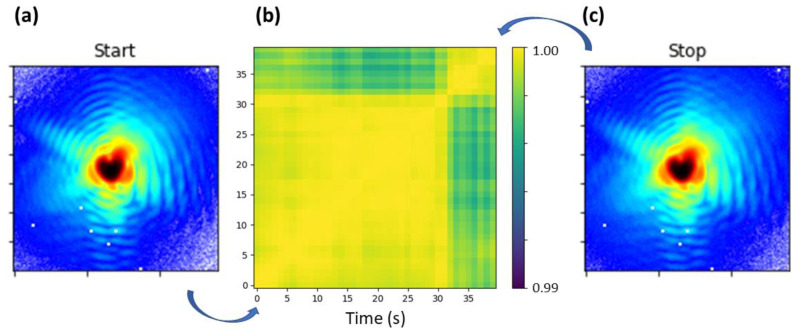
Cross-correlation of the scattered intensity when increasing load from 0.5 µN to 1 µN. (**a**) Slice of the 111 Bragg peak taken at the maximum when starting indentation. (**b**) Cross-correlation of the intensity during loading. (**c**) Slice of the 111 Bragg peak taken at the maximum when stopping indentation.

The cross-correlation of the intensity measured at the Au **111** Bragg reflection during loading is reported in Figure 2. It demonstrates that the intensity does not evolve continuously but with rapid changes. Indeed, no variations are apparent during the first 30 s while rapid changes of the intensity distribution evidence a modification of the nanostructure of the crystal.

**Figure 3:**
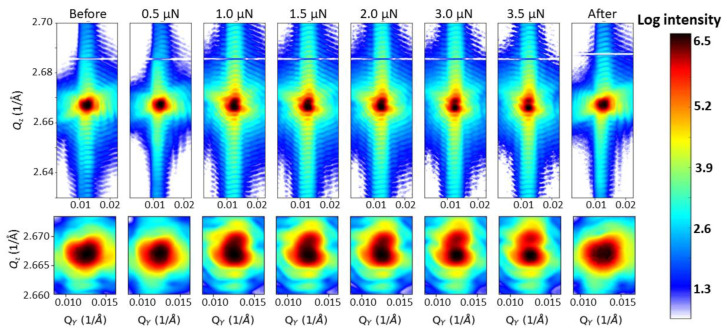
In situ mw-BCDPs integrated along *Q*_x_ of the Au 111 Brag reflection recorded during the nano-indentation of a strain-free and defect-free Au crystal. The lower row shows a closeup of the central part of the Au 111 Bragg peak shown in the upper row.

Mw-BCDPs were recorded at 6 different loads up to a maximum force of *F* = 3.5 µN as well as after complete unloading. Figure 3 shows the intensity in the *Q*_z_-*Q*_y_ plane integrated over 40 sections coming from the interpolated 3D diffraction peaks for each force value as well as before contact and after retraction of the SFINX-tip. While the core of the Bragg reflection of the initial crystal is homogeneous in intensity, its intensity gradually decreases, it becomes elongated and finally divides into two parts, creating two distinct peaks. The zoom-in on the core of the Bragg peak displayed in the lower row of Figure 3 clearly shows the evolution of the core of the Bragg reflection which regains its homogeneity after retraction of the SFINX-tip. It is fully reversible, demonstrating a pseudo-elastic behavior that can only be visualized by in situ experiments while it is invisible for *post-mortem* studies. 

**Figure 4:**
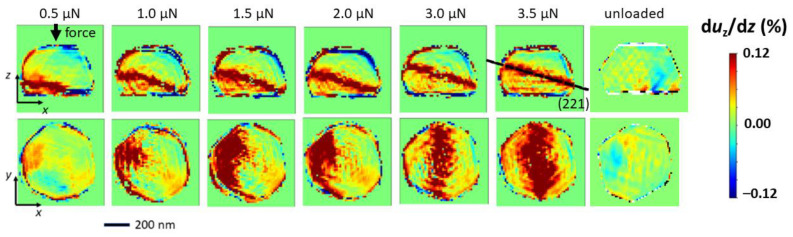
Reconstructed out-of-plane strain field in the Au crystal during nano-indentation reconstructed from the BCDPs presented in Figure 2. *X*, *Y*, and *Z* axis correspond to the $\left[11\bar{2}\right]$, $\left[\bar{1}10\right]$ and [111] crystallographic directions, respectively.

The Bragg electron density and the phase were reconstructed using the PyNX software package [23]. Here, the *X, Y,* and *Z* axis correspond to the $\left[11\bar{2}\right]$, $\left[\bar{1}10\right]$ and [111] crystallographic directions, respectively. From the phase the deformation along the z-direction was inferred which is illustrated in Figure 4 for the different applied loads. The out-of-plane deformation varies in a range of ±0.12% within the crystal with a band of deformation actually dividing the crystal into two parts where the upper part is in compression with respect to the lower part. It is worth noting that the zero-strain value was fixed arbitrarily. The band of deformation starts at the lower left border of the crystal close to the crystal-substrate interface and propagates towards the crystal center with increasing applied mechanical load. After complete unloading of the crystal, it is actually strain-free and defect-free confirming the pseudo-elastic behavior already indicated by the variation of the Bragg reflection and its return to its initial shape and structure (see Figure 3).

The cross-correlation of the 3D intensity distribution measured before loading, at the different loading steps as well as after complete unloading, is presented in Figure S1 of the Supplementary Material. While the value of the cross-correlation decreases to 0.95 for the first loading step of 0.5 µN and diminishes further to <0.8 for the highest applied force of 3.5 µN, it amounts to about 0.98 after complete unloading. Although the cross-correlation is not exactly 1.00, the small difference confirms the pseudo-elastic behavior of the particle.

## 4. Discussion

The band of deformation, which is oriented along the (221) crystal plane, might actually originate from a shearing of the crystal due to an unintentional off-centered application of the force on the crystal. In addition, possible wearing out of the AFM-tip during the AFM imaging for sample alignment may have blunted the AFM-tip or particles that could have been attached to the AFM-tip, leading to a non-ideal nanoindentation. Considering a load application on one of the inclined facets instead of the crystal top facet induces a torque, thus leading to a shearing of the particle. An additional torque may be induced by the bending of the AFM cantilever during loading, which will inevitably result in a non-vertical force application during the indentation process, intensifying the crystal shearing. Considering that dewetted particles, as they are used in the present work, typically exhibit dislocations at the particle-substrate interface, the unintentional shearing will lead to the nucleation of defects at this interface, which may propagate upwards towards the crystal center with an increasing applied load depending on their respective Burger’s vectors.

The (221) shear band observed in the present study deviates from the {111} slip or twinning planes typical for the *fcc* Au. The slip traces corresponding to the {111} slip planes have been observed in faceted single crystalline particles of Pt [24] and of Ni-Au alloys [25] compressed by a flat diamond punch. The fact that the highly strained planar feature observed in the present work deviates from crystallographic {111} plane indicates that this is most likely a highly localized shear band, since the shear bands in *fcc* metals do not usually follow the low-index crystallographic planes [26]. The localized shear bands contain a high density of dislocations, stacking faults, and other defects, leading to high strain localization. In bulk crystals, the deformation via shear banding is irreversible. The striking phenomenon of the reversible strain evolution observed in the present study indicates that shear bands formed in small sub-micrometer size crystals can fully recover after unloading. This recovery is probably associated with reversible dislocations nucleation/annihilation at the side surface of the particle and at the particle–substrate interface. It should be noted that reversible dislocation-mediated pseudo-elastic deformation during the formation of adhesive contact between two faceted Au nanoparticles has been predicted in atomistic simulations of Mordehai et al. [27]. A full recovery of initial defect- and strain-free microstructure of the particle upon unloading sheds new light on the extraordinary mechanical properties of metal nanoparticles obtained by solid-state dewetting. Indeed, reversible deformations exceeding 10% have been observed in compression tests of Au [28], Ni [29], and Pt [24] nanoparticles. According to the results of the present work, these high compressive deformations may in fact be pseudo-elastic, and the stochastic nature of the nucleation of shear bands can explain the high scatter of slopes of the respective sections of the load-displacement curves.

It should be noted that the detector response becomes non-linear for high diffraction intensities, as in the present study. Such non-linearities were corrected for by renormalization of the detector response. However, they may affect the actual phase retrieval process. Regardless, the splitting of the Bragg peak core as well as its reversibility are undoubtedly independent of a possible non-linear detector response. It is further worth mentioning that the measurement of one mw-BCDI took about 30 min in comparison to about 10 min for standard BCDI by rocking the sample. The longer measurement time is due to the adjustment of the undulator gaps to maintain a constant incident X-ray intensity while scanning the X-ray energy. During this time, dislocations and defects diffuse within the crystal; thus, individual dislocations or nucleation events could not be visualized. In any case, while previous nano-indentation experiments combined with BCDI were only able to image the microstructure of the crystal before loading and after complete unloading, the present work shows for the first time the evolution of the microstructure under an externally applied mechanical load. As presented here, the crystal is completely free of defects once the external stimulus is released; thus, *post-mortem* imaging would not be able to catch this kind of deformation mechanism in sub-micrometric crystals in which defects are attracted to free surfaces wherein they are annihilated.

It must be borne in mind that one single Bragg peak only provides access to the deformation along one single *Q*-vector, thus limiting the information of the actual three-dimensional strain field of an object. In order to measure the deformation along several crystallographic directions and eventually obtain the complete strain tensor of a nanostructure, at least three independent Bragg peaks must be measured. The measurement of several Bragg peaks does not pose major problems for ex situ BCDI experiments. However, in situ BCDI experiments are so far restricted to single Bragg reflections and are thus limited regarding the displacement field and the detection and identification of defects due to the G·b criterion, i.e., that dislocations with the Burger’s vector *b* being perpendicular to the scattering vector *G* (G·b=0) are actually invisible. In the case of reversible deformations, it is possible to measure several Bragg peaks consecutively in consecutive cycles of external stimuli, as demonstrated during hydrogen peroxide decomposition on Pt nanoparticles [30]. For imaging irreversible changes such as plasticity (such as in the case of the present work) and the nucleation and evolution of defects and dislocation networks, consecutive measurements capture different deformation states of the structure under study. Future studies will focus on the simultaneous in situ measurement of at least two Bragg reflections at the same time, as it has been demonstrated for a twinned Au crystal [31].

## 5. Conclusions

In conclusion, the first non-destructive in situ 3D imaging of both the microstructure and dislocations in a sub-micrometric crystal during nano-indentation is reported. A (221) shear band was nucleated at the left lower corner of the Au crystal close to the crystal-substrate interfaces and propagates towards the crystal center with an increasing applied mechanical load. Once the mechanical load was removed, the dislocations diffuse to the free surfaces, where they are annihilated. The complete recovery of the initial strain-free and defect-free Au crystal illustrates the extraordinary mechanical properties of crystals obtained by solid-state dewetting. While standard BCDI does not allow for following the mechanical deformation during indentation but only after releasing the mechanical stimulus due to detrimental vibrations induced by diffractometer motor movements during rocking scans, we demonstrate here the monitoring of the evolution of the microstructure of a sub-micrometric crystal in 3D by mw-BCDI during nano-indentation. Measurement of several independent Bragg peaks will allow for reconstructing the complete strain tensor and to image all dislocations existing in the indented crystal. With the current upgrade of the third-generation synchrotron sources to extremely brilliant sources, quicker measurements with higher spatial resolution will be feasible in the future, making the measurement of several non-coplanar Bragg reflections possible in a reasonable time without risking the stability of nano-mechanical testing setups.

## Data Availability

The data that support the findings of this study are available within the article and its supplementary material.

## Supplementary Materials

The following supporting information can be downloaded at: https://www.mdpi.com/article/10.3390/ma15186195/s1, Details about the multi-wavelength Bragg coherent X-ray diffraction and the phase retrieval. Cross-correlation of the mw-BCDPs measured before loading, at different loading steps, and after complete unloading. References [32,33,34,35,36,37] are cited in the supplementary materials.

## References

[1] Herring C., Galt J.K. (1952). Elastic and plastic properties of very small metal specimens. Phys. Rev..

[2] Brenner S.S. (1956). Tensile strength of whiskers. J. Appl. Phys..

[3] Uchic M.D., Dimiduk D.M., Florando J.N., Nix W.D. (2004). Sample dimensions influence strength and crystal plasticity. Science.

[4] Uchic M.D., Shade P.A., Dimiduk D.M. (2009). Plasticity of micrometer-scale single crystal in compression. Annu. Rev. Mater. Res..

[5] Kiener D., Grosinger W., Dehm G., Pippan R. (2008). A further step towards an understanding of size-dependent crystal plasticity: In situ tension experiments of miniaturized single-crystal copper samples. Acta Mater..

[6] Maaß R., Van Petegem S., Van Swygenhoven H., Derlet P.M., Volkert C.A., Grolimund D. (2007). Time-resolved Laue diffraction of deforming micropillars. Phys. Rev. Lett..

[7] Kirchlechner C., Keckes J., Micha J.-S., Dehm G. (2011). In situ µLaue: Instrumental setup for the deformation of micron sized samples. Adv. Eng. Mat..

[8] Lee S., Im J., Yoo Y., Bitzek E., Kiener D., Richter G., Kim B., Oh S.H. (2014). Reversible cyclic deformation mechanism of gold nanowires by twinning-detwinning transition evidenced from in situ TEM. Nat. Commun..

[9] Sun J., He L., Lo Y.-C., Xu T., Bi H., Sun L., Zhang Z., Mao S.X., Li J. (2014). Liquid-like pseudoelasticity of sub-10-nm crystalline silver particles. Nat. Mater..

[10] Dehm G., Jaya B.N., Raghavan R., Kirchlechner C. (2018). Overview on micro- and nanomechanical testing: New insights in interface plasticity and fracture at small length scales. Acta Mater..

[11] Cha W., Ulvestad A., Allain M., Chamard V., Harder R., Leake S.J., Maser J., Fuoss P.H., Hruszekewycz S.O. (2016). Three-dimensional variable-wavelength X-ray Bragg coherent diffraction imaging. Phys. Rev. Lett..

[12] Lauraux F., Cornelius T.W., Labat S., Richard M.-I., Leake S., Zhou T., Kovalenko O., Rabkin E., Schülli T.U., Thomas O. (2020). Multi-wavelength Bragg coherent X-ray diffraction imaging of Au particles. J. Appl. Crystallogr..

[13] Ren Z., Mastropietro F., Langlais S., Davydok A., Richard M.-I., Thomas O., Dupraz M., Verdier M., Beutier G., Boesecke P. (2014). Scanning force microscopy for in situ nanofocused X-ray diffraction studies. J. Synchrotron Radiat..

[14] Dupraz M., Beutier G., Cornelius T.W., Parry G., Ren Z., Labat S., Richard M.-I., Chahine G.A., Kovalenko O., Rabkin E. (2017). 3D imaging of a dislocation loop at the onset of plasticity in an indented nanocrystal. Nano Lett..

[15] Robinson I., Harder R. (2009). Coherent X-Ray Diffraction Imaging of Strain at the Nanoscale. Nat. Mater..

[16] Dupraz M., Beutier G., Rodney D., Mordehai D., Verdier M. (2015). Signature of dislocations and stacking faults of face-centred cubic nanocrystals in coherent X-ray diffraction patterns: A numerical study. J. Appl. Crystallogr..

[17] Cornelius T.W., Davydok A., Jacques V.L.R., Grifone R., Schülli T., Richard M.-I., Beutier G., Verdier M., Metzger T.H., Pietsch U. (2012). In situ 3D reciprocal space mapping during mechanical deformation. J. Synchrotron Radiat..

[18] Davydok A., Cornelius T.W., Ren Z., Leclere C., Chahine G., Schülli T., Lauraux F., Richter G., Thomas O. (2018). In Situ Coherent X-ray Diffraction during Three-Point Bending of a Au Nanowire: Visualization and Quantification. Quantum Beam Sci..

[19] Kovalenko O., Greer J.R., Rabkin E. (2013). Solid-state dewetting of thin iron films on sapphire substrates controlled by grain boundary diffusion. Acta Mater..

[20] Leake S.J., Chahine G.A., Djazouli H., Zhou T., Richter C., Hilhorst J., Petit L., Richard M.-I., Morawe C., Barrett R. (2019). The nanodiffraction beamline ID01/ESRF: A microscope for imaging strain and structure. J. Synchrotron Radiat..

[21] Chahine G.A., Richard M.-I., Homs-Regojo R.A., Tran-Caliste T.N., Carbone D., Jacques V.L.R., Grifone R., Boesecke P., Katzer J., Costina I. (2014). Imaging of strain and lattice orientation by quick scanning X-ray microscopy combined with three-dimensional reciprocal space mapping. J. Appl. Crystallogr..

[22] Chahine G.A., Zoellner M.H., Richard M.-I., Guha S., Reich C., Zaumseil P., Capellini G., Schroeder T., Schülli T.U. (2015). Lattice tilt and strain mapped by X-ray scanning nanodiffraction in compositionally graded SiGe/Si microcrystals. Appl. Phys. Lett..

[23] Favre-Nicolin V., Girard G., Leake S., Carnis J., Chushkin Y., Kieffer J., Paleo P., Richard M.-I. (2020). PyNX: High-Performance Computing Toolkit for Coherent X-Ray Imaging Based on Operators. J. Appl. Crystallogr..

[24] Zimmerman J., Bisht A., Mishin Y., Rabkin E. (2021). Size and shape effect on the strength of platinum nanoparticles. J. Mater. Sci..

[25] Herre P., Will J., Dierner M., Wang D., Yokosawa T., Zech T., Wu M., Przybilla T., Romeis S., Unruh T. (2021). Rapid fabrication and interface structure of highly faceted epitaxial Ni-Au solid solution nanoparticles on sapphire. Acta Mater..

[26] Jia N., Eisenlohr P., Roters F., Raabe D., Zhao X. (2012). Orientation dependence of shear banding in face-centered-cubic single crystals. Acta Mater..

[27] Mordehai D., Rabkin E., Srolovitz D.J. (2011). Pseudoelastic deformation during nanoscale adhesive contact formation. Phys. Rev. Lett..

[28] Mordehai D., Lee S.-W., Backes B., Srolovitz D.J., Nix W.D., Rabkin E. (2011). Size effect in compression of single-crystal gold microparticles. Acta Mater..

[29] Sharma A., Hickman J., Gazit N., Rabkin E., Mishin Y. (2018). Nickel nanoparticles set a new record of strength. Nat. Commun..

[30] Choi S., Chung M., Kim D., Kim S., Yun K., Cha W., Harder R., Kawaguchi T., Liu Y., Ulvestad A. (2020). In situ strain evolution on Pt nanoparticles during hydrogen peroxide decomposition. Nano Lett..

[31] Lauraux F., Labat S., Yehya S., Richard M.-I., Leake S., Zhou T., Micha J.-S., Robach O., Kovalenko O., Rabkin E. (2021). Simultaneous multi-Bragg peak coherent X-ray diffraction imaging. Crystals.

[32] Leake S.J., Favre-Nicolin V., Zatterin E., Richard M.-I., Fernandez S., Chahine G., Zhou T., Boesecke P., Djazouli H., Schülli T.U. (2017). Coherent nanoscale X-ray probe for crystal interrogation at ID01, ESRF–The European Synchrotron. Mater. Des..

[33] Mandula O., Elzo Aizarna M., Eymery J., Burghammer M., Favre-Nicolin V. (2016). PyNX.Ptycho: A computing library for X-ray coherent diffraction imaging of nanostructures. J. Appl. Cryst..

[34] Ponchut C., Rigal J.M., Clément J., Papillon E., Homs A., Petitdemange S. (2011). MAXIPIX, a fast readout photon-counting X-ray area detector for synchrotron applications. J. Instr..

[35] Luke D.R. (2004). Relaxed averaged alternating reflections for diffraction imaging. Inverse Probl..

[36] Fienup J.R. (1978). Reconstruction of an object from the modulus of its Fourier transform. Opt. Lett..

[37] Gerchberg R.W., Saxton W.O. (1972). A practical algorithm for the determination of the phase from image and diffraction plane pictures. Optik.

