# Supplementary material for: In Situ Nano-Indentation of a Gold Sub-Micrometric Particle Imaged by Multi-Wavelength Bragg Coherent X-ray Diffraction
Source: Materials (Basel). 2022 Sep 6;15(18):6195. doi: 10.3390/ma15186195 (PMC9501309; doi:10.3390/ma15186195)
Supplement: Supplementary file 1 [file materials-15-06195-s001.zip › materials-1870371-Supplementary.pdf]

materials MDPI

Supplementary Material

In situ nano-indentation of a gold sub-micrometric particle imaged by multi-wavelength Bragg coherent X-ray diffraction

Florian Lauraux [1], Stéphane Labat [1], Marie-Ingrid Richard [1,2], Steven J. Leake [2], Tao Zhou [2,3], Oleg Kovalenko [4], Eugen Rabkin [4], Tobias U. Schülli [2], Olivier Thomas [1], and Thomas W. Cornelius [1,*]

[1] Aix Marseille Univ., Université de Toulon, CNRS, IM2NP, Marseille, France
[2] ID01/ESRF – The European Synchrotron, 71 Avenue des Martyrs, 38000, Grenoble, France
[3] Center for Nanoscale Materials, Argonne National Laboratory, 9700 S Cass Ave, Lemont, IL 60439, USA
[4] Department of Materials Science and Engineering, Technion – Israel Institute of Technology, 3200003 Haifa, Israel
* Correspondence: thomas.cornelius@im2np.fr

Multi-wavelength (mw) BCDI

Multi-wavelength Bragg coherent X-ray diffraction imaging of the specular Au **111** Bragg peak was performed at the ID01 beamline at the European Synchrotron ESRF in Grenoble (France). The incident X-ray photon energy was set to 9 keV using the Si (111) double-crystal monochromator. The incident X-ray beam was focused down using a pair of achromatic Kirkpatrick-Baez (KB) mirrors. A set of slits in front of the KB mirrors were closed down to 100 µm (H) x 300 µm (V) matching the lateral coherence lengths of the beamline and, thus selecting the coherent part of the incident X-ray beam. A pinhole of 50 µm in diameter was placed between the focusing optics and the sample, at 2 cm upstream from the sample position. The focal profile of the incident X-ray beam was characterized by 2D ptychography of a 30-µm diameter tungsten Siemens star placed close to the focal position of the KB mirrors [1,2]. Both the tungsten structure and the complex-valued wavefront were retrieved simultaneously using the ptychography reconstruction code of the PyNX package [3] resulting in a Gaussian shaped focal spot with a size of 550 and 530 nm in the vertical and in the horizontal direction, respectively. The diffracted X-rays were recorded using a 2D MAXIPIX pixel detector with a pixel size of 55 µm x 55 µm installed 1 m downstream from the sample position [4].

Mw-BCDI during nano-indentation of selected Au crystals was recorded by scanning the photon energy of the incident X-ray beam from 8.75 to 9.25 keV in steps of 2 eV and scanning simultaneously the 2θ angle of the detector by -/+ 1° in steps of 8 millidegrees. Every 5 energy steps the undulator gaps were readjusted to maintain the incident intensity I_0 constant all over the scanned energy range.

Phase retrieval

Both the Bragg electron density and the phase were reconstructed from the mw-BCDPs using the CDI algorithms in the PyNX package. A partial coherence point-spread function was employed to take into account the fact that the incident beam is not fully coherent. The intensity auto-correlation with a relative threshold of 0.1 was applied as initial support. The Hermitian symmetry of the complex scattered amplitude leads to the reconstruction of two conjugated objects that are equivalent. The support was cut in half during 10 cycles in order to bias the algorithm towards one solution. The support was updated every 20 cycles with a relative threshold of 0.18 and the absolute threshold was

obtained by averaging the values taken over the support volume. It was further updated by convoluting the object amplitude with a Gaussian with a size exponentially decreasing from 2 to 1. The algorithms used were first 800 RAAR (Relaxed Averaged Alternating Reflections) [5] followed by 150 ER (Error reduction [6,7]).

3D Cross-correlation

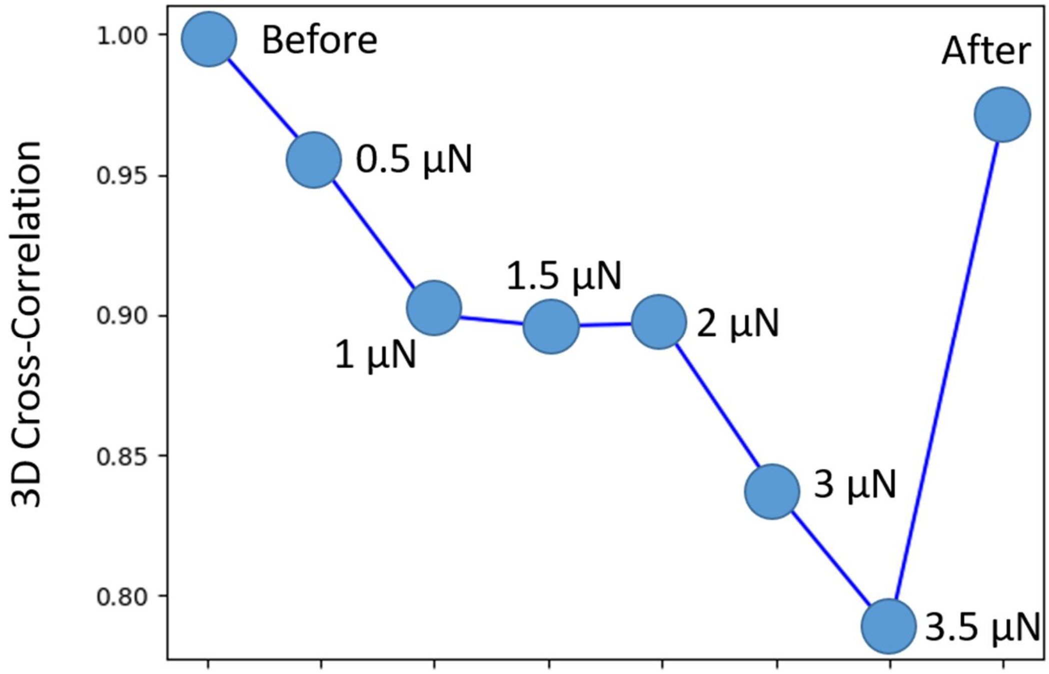

Figure S1: Cross-correlation of the 3D intensity distribution measured by mw-BCDI before loading, at the different loading steps, and after complete unloading.

References

1. Lauraux, F.; Cornelius, T.W.; Labat, S.; Richard, M.-I.; Leake, S.; Zhou, T.; Kovalenko, O.; Rabkin, E.; Schülli, T.U.; Thomas, O. Multi-wavelength Bragg coherent X-ray diffraction imaging of Au particles. *J. Appl. Cryst.* **2020**, *53*, 170-177
2. Leake, S.J.; Favre-Nicolin, V.; Zatterin, E.; Richard, M.-I.; Fernandez, S.; Chahine, G.; Zhou, T.; Boesecke, P.; Djazouli, H.; Schülli, T.U. Coherent nanoscale X-ray probe for crystal interrogation at ID01, ESRF – The European Synchrotron, *Materials & Design* **2017,** *119*, 470-471
3. Mandula, O.; Elzo Aizarna, M.; Eymery, J.; Burghammer, M.; Favre-Nicolin, V. PyNX.Ptycho: a computing library for X-ray coherent diffraction imaging of nanostructures, *J. Appl. Cryst.* **2016,** *49*, 1842.
4. Ponchut, C.; Rigal, J.M.; Clément, J.; Papillon, E.; Homs, A.; Petitdemange, S.; MAXIPIX, a fast readout photon-counting X-ray area detector for synchrotron applications, *J. Instr.* **2011,** *6*, C01069.
5. Luke, D.R. Relaxed averaged alternating reflections for diffraction imaging, *Inverse Problems* **2004,** *21*, 37–50.
6. Fienup, J.R. Reconstruction of an object from the modulus of its Fourier transform, *Optics Letters* **1978,** *3*, 27–29
7. Gerchberg, R.W.; Saxton, W.O. A practical algorithm for the determination of the phase from image and diffraction plane pictures, *Optik* **1972,** *35*, 237.
